# “Open-book” pelvic fracture with soft tissue serious damage in a child

**DOI:** 10.1007/s11751-015-0216-4

**Published:** 2015-03-07

**Authors:** Antonio Panella, Angela Notarnicola, Giuseppe Solarino, Biagio Moretti

**Affiliations:** Orthopaedic Units, Department of Medical Science of Base, Neuroscience and Organs of Sense, University of Bari, Piazza G.Cesare 11, 70124 Bari, Italy

**Keywords:** Open-book fractures, Child, Surgery, Skin lesions

## Abstract

Open-book fractures of the pelvis are uncommon during childhood and require urgent treatment from the association with other abdominal, vascular or nervous injuries. The case discussed is an open-book fracture (type B1, Tile classification) associated with triradiate cartilage injury (type I, Salter–Harris classification) in an 11-year-old female. Surgical treatment was delayed for 2 months due to an associated extensive cutaneous lesion which required an adequate treatment. The delayed intervention did not affect the radiological and clinical healing of the fracture.

## Introduction

Unstable pelvic fractures in children are uncommon. The incidence has been reported to be from 2.4 [1] to 7.5 % [2] of all children requiring admission after blunt trauma. The percentage of associated soft tissue lesions is very high [3].

## Case report

**Fig. 1:**
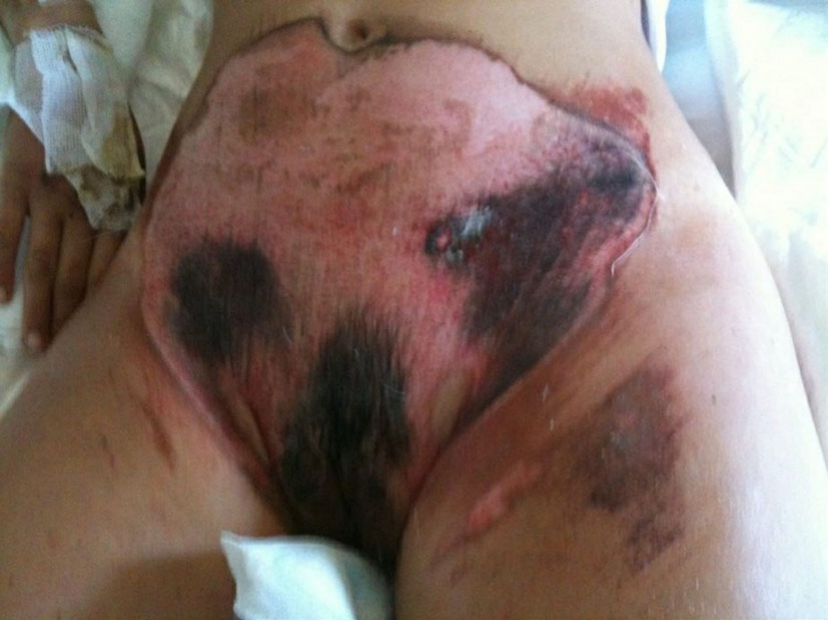
Wide skin lesion of abdomen and pelvis

**Fig. 2:**
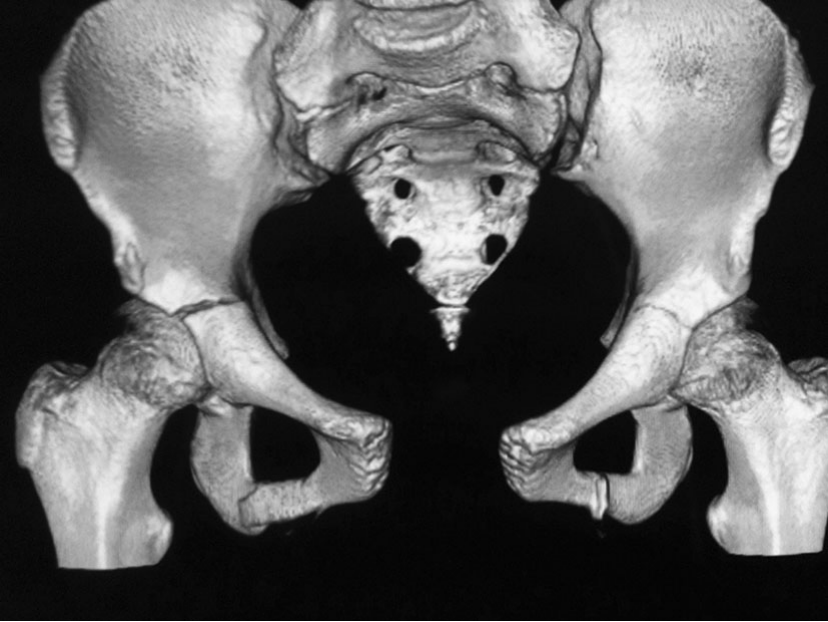
3D CT of pelvis: integrity of both sacroiliac joints, symphysis diastasis (Tile B1) and damage to triradiate cartilage at right (type I of Salter–Harris)

An 11-year-old girl presented to the emergency department after a motor vehicle accident. She was awake and alert (Glasgow Coma Scale score 15) and was found to have a wide (at least 600 cm^2^) and deep abdominal skin contusion (Fig. 1). The contusion involved both iliac crests. No vascular or neurological deficits were present. She complained of severe hip pain, made worse on standing. She underwent a CT (computed tomography) scan and had vascular or internal abdominal lesions excluded. The X-rays and the CT showed a 3.5-cm diastasis of the pubic symphysis (type B1, Tile classification) [4] with a right triradiate cartilage lesion (type I, Salter and Harris classification) [5] (Fig. 2). Due to the severity of the soft tissue contusion, a decision was made to postpone surgery [6–8]. The initial management of the diastasis was bed rest. Standard orthopaedic principles were observed insofar as the soft tissue management took precedence over the bony management [6–8]. Temporary external fixation was discounted because of a higher risk of infection [7]. After 20 days, the patient underwent surgery to cover the skin wound in the suprapubic area with a skin graft from the medial part of the thigh. After 60 days, the skin contusion was noted to be healing well and there was union of the triradiate cartilage injury without any major deformity. Definitive surgery, performed at 60 days, had particular attention made to displace the bladder which was adherent anteriorly to the pelvis. The interpubic fibro-cartilaginous lamina was removed. A surgical separation of the fracture ends was performed; it was noted that there was no excessive fibrous tissue between the two parts of the fracture. Two plates were used for a greater assurance of stability. Internal fixation of the symphysis was carried out using Matta plates (Stryker, USA) through a Pfannenstiel approach. The intraoperative X-rays showed a good reduction in the diastasis with satisfactory morphology of the acetabulum.

**Fig. 3:**
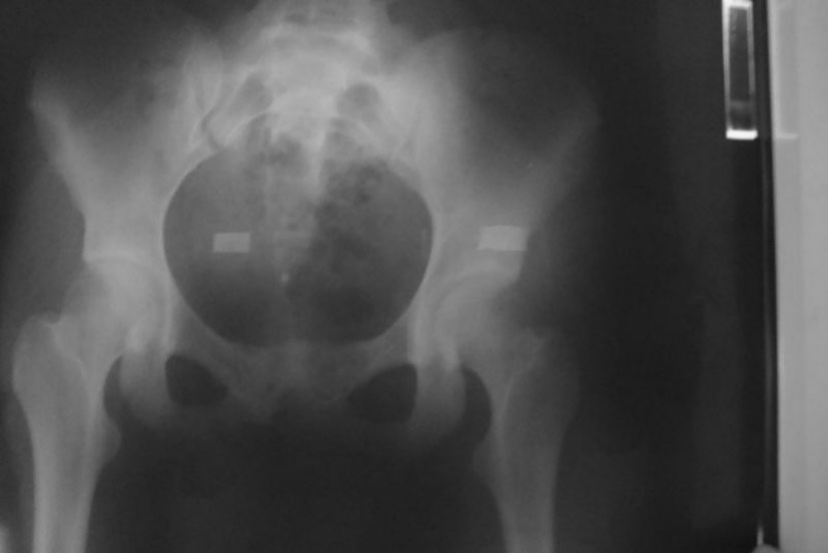
Post-operative X-ray following plate removal: maintained reduction in pubic symphysis with bone bridge and the closure of both triradiate cartilages

Mobilization was allowed the day after, and after 20 days, the patient was allowed to sit upright. Standing was permitted after 2 months. At 12-month follow-up, the clinical assessment showed the patient to be completely asymptomatic and without evidence of post-traumatic deformity of the hip. Implant removal was carried out 15 months after the initial injury (Fig. 3).

At the last follow-up, 20 months after surgical stabilization, the patient had no pain or functional restriction; the X-rays showed complete healing of the diastasis with total fusion of the triradiate cartilage bilaterally. The original skin lesion was healed completely. There was no leg-length discrepancy, and the patient had symmetric hip movement measuring greater than 120° flexion, 40° of internal rotation, 90° of external rotation and 60° of abduction.

## Discussion

Unstable pelvic ring fractures in childhood are uncommon (about 1:100,000) and are due to high-energy trauma [6]. The elastic characteristics of bone in childhood are the reason why this fracture is uncommon and why, when present, it is life-threatening [7]. The high-energy mechanism produces other lesions, e.g. vascular or visceral [9]. The injury to the triradiate cartilage is a risk for post-traumatic deformity of the hip [5]. The Tile classification is used widely and in paediatric injuries: type A are considered stable; type B present a vertical stability but rotational instability; and Type C present both vertical and rotational instability [4].

The case presented was a type B1 (open-book) fracture with a sagittal compression of the anterior arch and posterior opening of the sacroiliac joint: it is rare and is not previously reported in published series of paediatric pelvic fractures [9, 10]. The literature advocates correction and stabilization when the diastasis is over 1.5–2 cm [7, 9, 10].

To the best of our knowledge, this is the first reported case in which the skin condition prevented early surgical treatment of pelvic fracture. Despite surgical reduction and fixation delayed to 2 months after the injury, reduction in the symphyseal diastasis was maintained after the hardware removal. In these patients, damage to triradiate cartilage is a consequence of the trauma and has to be monitored for premature closure [5].

As the iliac bone is stabilized proximally by the sacroiliac joint and distally by the triradiate cartilage, severe trauma can lead to fracture and/or dislocation of both articulations in children, especially in type B1 fractures. The triradiate lesion may be a type I Salter–Harris injury with a better prognosis (as was in the one we treated) or a type II or V (with a worse prognosis). The surgical treatment of these lesions is debated, but internal fixation is widely considered a good option for triradiate cartilage lesions.

In the case presented, surgical treatment to the triradiate cartilage injury was deferred as it was deemed stable; 2 months after injury, additional surgical trauma may have had a detrimental effect on the cartilage closure as previously reported [10]. On the other hand, it has been indicated that the patient’s age at the time of injury is very important for the prognosis of triradiate cartilage injuries. According to Bucholz et al. [5], severe deformity has not been reported after the age of 10–11 years. The X-rays at the final follow-up supported our decision not to operate on the triradiate cartilage as no sign of premature closure of the triradiate cartilage was seen. The factors responsible for the favourable outcome are likely to be the following: the type of lesion (Salter–Harris I lesion), age (11 years) and the decision not to treat the triradiate lesion.

## Conclusion

This case report highlights a delayed approach to surgical treatment of an unstable pelvic fracture owing to severe and unsuitable soft tissue conditions precluding immediate surgery. It shows that this approach is compatible with a favourable outcome despite injuries to the triradiate cartilage and symphysis pubis.
